# Erratum to: Hypoxia alters vulnerability to capture and the potential for trait-based selection in a scaled-down trawl fishery

**DOI:** 10.1093/conphys/coz111

**Published:** 2020-01-17

**Authors:** Davide Thambithurai, Amelie Crespel, Tommy Norin, Anita Rácz, Jan Lindström, Kevin J Parsons, Shaun S Killen

**Affiliations:** 1 Institute of Biodiversity, Animal Health and Comparative Medicine, University of Glasgow, Graham Kerr Building, Glasgow G12 8QQ, UK; 2 DTU Aqua: National Institute of Aquatic Resources, Technical University of Denmark, Kemitorvet, Building 202, 2800 Kgs. Lyngby, Denmark; 3 Department of Genetics, Eötvös Loránd University, Pázmány P.s. 1C, H-1117 Budapest, Hungary

*Conserv Physiol* 7(1): coz082; doi: 10.1093/conphys/coz082

An earlier version of this manuscript, which had not been approved by the author, was inadvertently published and therefore contained several minor grammatical and formatting errors. The final version of this manuscript has now replaced this earlier version, and so these errors are no longer present. The Publisher apologises for this mistake, and any confusion caused.

